# The impact of primary dysmenorrhea on adolescents’ activities and school attendance

**DOI:** 10.25122/jml-2023-0247

**Published:** 2023-10

**Authors:** Ainur Donayeva, Ainur Amanzholkyzy, Ibrahim Abdelazim, Roza Nurgaliyeva, Gulnara Gubasheva, Samat Saparbayev, Dinmukhamed Ayaganov, Aiman Kaldybayeva, Ihab Samaha

**Affiliations:** 1 Department of Normal Physiology, West Kazakhstan Marat Ospanov Medical University, Aktobe, Kazakhstan; 2 Department of Obstetrics and Gynecology, Faculty of Medicine, Ain Shams University, Cairo, Egypt; 3 Department of Obstetrics and Gynecology №2, West Kazakhstan Marat Ospanov Medical University, Aktobe, Kazakhstan; 4 Department of Neurology, West Kazakhstan Marat Ospanov Medical University, Aktobe, Kazakhstan; 5 Department of Obstetrics and Gynecology, Faculty of Medicine, Helwan University, Cairo, Egypt

**Keywords:** activities, adolescents, dysmenorrhea, absenteeism, BMI: Body Mass Index, HSD: Honestly Significant Difference, NSAIDs: Nonsteroidal Anti-Inflammatory Drugs, OR: Odds Ratio, PD: Primary Dysmenorrhea, RK: Republic of Kazakhstan, SPSS: Statistical Package for the Social Sciences, VAS: Visual Analog Scale, Vit. D: Vitamin D, WHO: World Health Organization, WK: West Kazakhstan, WKMU: West Kazakhstan Medical University

## Abstract

Primary dysmenorrhea is the most commonly encountered menstrual issue among adolescents, often leading to significant school absenteeism. This study aimed to detect the impact of primary dysmenorrhea on adolescents’ activities and school attendance. We conducted a cross-sectional comparative study involving 180 adolescents aged 12 to 18 who experienced primary dysmenorrhea. A comprehensive trans-abdominal pelvic sonography was performed to rule out any underlying pelvic conditions. The severity of dysmenorrhea was evaluated using the visual analog scale (VAS), categorizing adolescents into groups with mild dysmenorrhea (VAS ≥1 to ≤3), moderate dysmenorrhea (VAS >3 to ≤7), and severe dysmenorrhea (VAS >7 to ≤10). Adolescents were surveyed to determine whether the severity of dysmenorrhea had an adverse effect on their physical and social activities as well as their school attendance. We used one-way ANOVA to compare the groups. There was a significant positive relation between the severity of dysmenorrhea and its negative impact on adolescents’ physical activities (r=0.395; p<0.00001) and social activities (r=0.658; p<0.00001). Additionally, there was a significant positive relation between the severity of dysmenorrhea and its negative impact on adolescents’ school attendance (r=0.416; p<0.00001). The odds of a negative impact on adolescents’ physical and social activities and school attendance were significantly higher in adolescents experiencing moderate and severe dysmenorrhea than in adolescents with mild dysmenorrhea.

## INTRODUCTION

Primary dysmenorrhea (PD) is characterized by painful lower abdominal cramps that typically occur just before menstruation and can last 8 to 72 hours after menstruation without any pelvic pathology [1]. Typically, PD begins either with the onset of menstruation or within 6-24 months after that, often accompanied by systemic symptoms such as headaches, fatigue, vomiting, and diarrhea [2]. It affects a substantial percentage of reproductive-age women, ranging from 16% to 91% [3]. The principal theory attributes PD to increased local prostaglandins, resulting in heightened uterine contractions and endometrial ischemia [4-6].

PD is the most prevalent menstrual problem in adolescence and the most common cause of school absenteeism [7]. Dysmenorrhea poses a significant public health concern as it can adversely affect work productivity [8]. Improving the quality of life for the younger generation and focusing on early disease prevention and treatment are crucial priorities within the Republic of Kazakhstan's healthcare program for 2020-2025 [9]. Therefore, this study aimed to detect the impact of PD on adolescents’ activities and school attendance, based on the hypothesis that PD is the most prevalent menstrual concern during adolescence and a leading factor contributing to school absenteeism among adolescents [7].

## MATERIAL AND METHODS

### Study design and participants

This cross-sectional comparative study included 180 adolescents aged 12 to 18, all diagnosed with primary dysmenorrhea. The study adhered to the Strengthening the Reporting of Observational Studies in Epidemiology (STROBE) guidelines for reporting observational research. Participants were recruited from 43 schools in West Kazakhstan (Aktobe) between 2021 and 2022, following ethical approval.

### Inclusion and exclusion criteria

The study included adolescents aged 12 to 18 years, with a normal body mass index (BMI) falling within the range of >18.5 - 24.9 kg/m^2^, regular menstrual cycle occurring every 21 to 35 days, and who experienced primary dysmenorrhea (defined as a visual analog scale (VAS) score ≥1) for a minimum of one year since menarche. Exclusion criteria included adolescents with pelvic organ anomalies (including genital and urinary tract anomalies), pelvic pathology (such as fibroid uterus or ovarian cyst or mass), a history of pelvic surgery, neurological or psychiatric disorders, or recent exogenous hormonal therapy within the past year.

### Data collection

Adolescents and their parents or guardians were provided with a clear definition of PD by RN and GG, both authors and researchers with over five years of experience in the field. Participants were included in the study after obtaining informed consent from the adolescents and their parents or guardians. Data collection was conducted through individual interviews between the participants and researchers AD and AA, both authors and researchers with more than five years of experience in the field, specializing in qualitative data collection. These interviews were held in private, confidential settings, which included private rooms within the adolescents' schools or at the diagnostic health center of WKMU.

### Clinical assessment

Adolescents underwent a comprehensive clinical assessment conducted by a gynecologist at the diagnostic health center of WKMU in accordance with the West Kazakhstan hospital's protocol. This assessment included a thorough medical history, measurement of weight and height to calculate BMI, and trans-abdominal pelvic sonography by an expert sonographer, blinded to the adolescents' data, to rule out pelvic pathology. The World Health Organization (WHO) classifies a BMI of 18.5-24.9 kg/m^2^ as normal, 25-29.9 kg/m^2^ as overweight, and >30 kg/m^2^ as obesity class-I [10, 11].

### Dysmenorrhea severity

**Figure 1:**
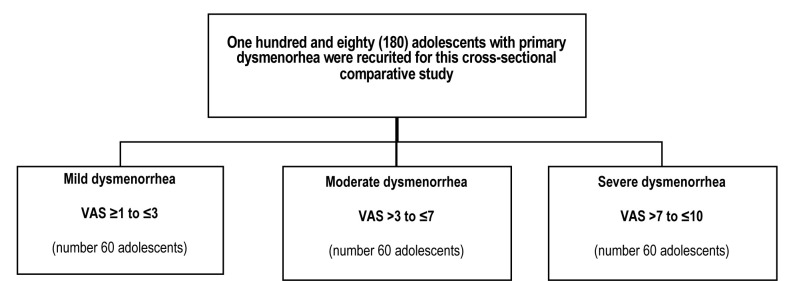
Study flowchart

The severity of dysmenorrhea was assessed using the visual analog scale (VAS), ranging from 0 (indicating no pain) to 10 (indicating unbearable pain) [12]. Adolescents were categorized based on their VAS scores into three groups: mild dysmenorrhea (VAS ≥1 to ≤3), moderate dysmenorrhea (VAS >3 to ≤7), and severe dysmenorrhea (VAS >7 to ≤10) (Figure 1) [13]. The adolescents were questioned about how the severity of their dysmenorrhea, classified as mild (VAS ≥1 to ≤3), moderate (VAS >3 to ≤7), or severe (VAS >7 to ≤10), impacted their engagement in physical and social activities, and their school attendance [14].

Physical activities refer to regular exercise and workouts as part of their daily routines. Social activities included family and school activities, including gatherings and celebrations. The participants' school absenteeism was determined through self-reporting and confirmed using the school's attendance records.

### Data analysis

The data collected were analyzed using a one-way analysis of variance test (ANOVA), followed by post-hoc Tukey Honestly Significant Difference (HSD) tests for group comparisons. Pearson's correlation coefficient (r) was used to assess the relationship between dysmenorrhea severity and its impact on adolescents' activities and school attendance. The MedCalc 20.106 software (MedCalc. Ltd., Belgium) was used to determine the odds of PD negatively affecting the activities and school attendance of the adolescents, which served as the primary outcome measure. Statistical significance was defined as p<0.05. The required sample size for this study was determined based on the number of adolescents aged 12-18 years in West Kazakhstan (27,972), the prevalence of dysmenorrhea among adolescents (ranging from 8% to 83%), a probability of 0.05, a power of 0.95, a sample size of 0.5. The sample size was calculated using G Power 3.1.9.7 software (Düsseldorf; Germany).

## RESULTS

### Participants characteristics

A number of 180 adolescents aged 12-18 years diagnosed with primary dysmenorrhea participated in this cross-sectional comparative study. They were categorized into three groups based on their VAS scores: mild dysmenorrhea (VAS ≥1 to ≤3), moderate dysmenorrhea (VAS >3 to ≤7), and severe dysmenorrhea (VAS >7 to ≤10), with the main outcome being the impact of PD on adolescents' activities and school attendance.

There were no significant differences in mean age and body mass index (BMI) among the three studied groups. The mild dysmenorrhea group had a mean age of 14.8±1.4 years and a BMI of 23.6±0.7 kg/m^2^. The moderate dysmenorrhea group had a mean age of 14.8±1.6 years and a BMI of 23.3±0.9 kg/m^2^, while the severe dysmenorrhea group had a mean age of 15.3±1.5 years and a BMI of 23.6±0.7 kg/m^2^ (Table 1). The categorization of participants into the three dysmenorrhea severity groups resulted in significant differences in VAS scores. Specifically, the mild dysmenorrhea group had a VAS score of 1.72±0.7, the moderate dysmenorrhea group had a VAS score of 5.02±0.9, and the severe dysmenorrhea group had a VAS score of 8.9±0.7 (Table 1).

**Table 1:** ANOVA analysis of group characteristics based on dysmenorrhea severity

Variables	N	Mean±SD	SS	DF	MS	F	Q_.05_=3.3426 & Q_.01_=4.1740 (p-value)
**Age (Years)** - Mild dysmenorrhea group - Moderate dysmenorrhea group - Severe dysmenorrhea group	60 60 60	14.8±1.4 14.8±1.6 15.3±1.5	11.025	2	5.5125	2.55682	G1:G2; Q=0.0 (p=0.0) G1:G3; Q=2.77 (p=0.13) G2:G3; Q=2.77 (p=0.13)
**Height (Cm)** - Mild dysmenorrhea group - Moderate dysmenorrhea group - Severe dysmenorrhea group	60 60 60	157.7±1.6 157.6±2.2 158.0±1.4	5.0333	2	2.5167	0.82565	G1:G2; Q=0.3 (p=0.98) G1:G3; Q=1.4 (p=0.58) G2:G3; Q=1.7 (p=0.45)
**Weight (Kg)** - Mild dysmenorrhea group - Moderate dysmenorrhea group - Severe dysmenorrhea group	60 60 60	58.7±2.1 58.0±2.8 58.8±2.1	22.536	2	11.2681	2.03485	G1:G2; Q=2.22 (p=0.26) G1:G3; Q=0.44 (p=0.95) G2:G3; Q=2.66 (p=0.15)
**BMI (Kg/m^2^)** - Mild dysmenorrhea group - Moderate dysmenorrhea group - Severe dysmenorrhea group	60 60 60	23.6±0.7 23.3±0.9 23.6±0.7	2.6888	2	1.3444	2.2044	G1:G2; Q=2.6 (p=0.16) G1:G3; Q=0.08 (p=0.99) G2:G3; Q=2.5 (p=0.18)
**VAS** - Mild dysmenorrhea group - Moderate dysmenorrhea group - Severe dysmenorrhea group	60 60 60	1.72±0.7 5.02±0.9 8.9±0.7	1566.21	2	783.106	1347.033	G1:G2; Q=33.5 (p=0.000001*) G1:G3; Q=73.3 (p=0.000001*) G2:G3; Q=39.8 (p=0.000001*)

* : Significant difference. BMI: Body mass index. Data presented as mean±SD (Standard Deviation). DF: Difference of freedom between groups. F: ANOVA analysis (ANOVA coefficient). MS: Mean of squares between groups. N: Number. A one-way ANOVA test was used to analyze variance between the studied groups. SD: Standard deviation. Significant value <0.05 according to post-hoc Tukey HSD (HSD.05=0.3290 and HSD.01=0.4109). SS: Sum of squares between groups. VAS: Visual Analogue Scale.

### Relationship between the severity of dysmenorrhea and its negative impacts

**Figure 2:**
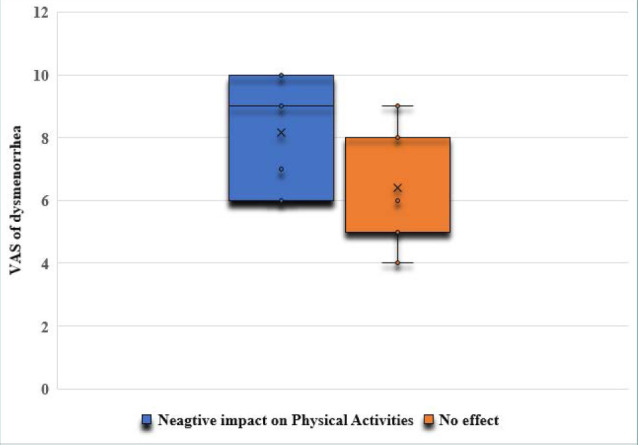
The impact of dysmenorrhea on adolescents’ physical activities

**Figure 3:**
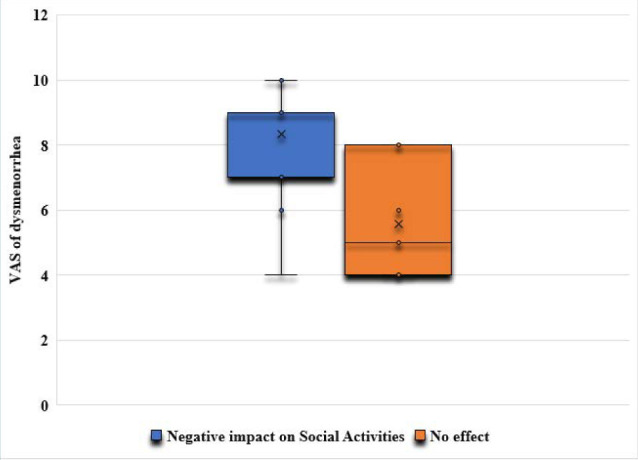
The impact of dysmenorrhea on adolescents’ social activities

**Figure 4:**
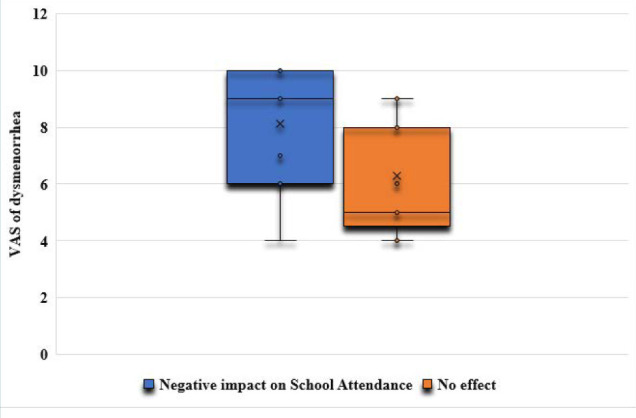
The impact of dysmenorrhea on adolescents’ school attendance

The correlation analysis showed a significant positive relationship between the severity of dysmenorrhea and its negative impact on adolescents’ physical activities (r=0.395; p<0.00001) (Figure 2) and social activities (r=0.658; p<0.00001) (Figure 3). Additionally, the correlation analysis showed a significant positive relationship between the severity of dysmenorrhea and its negative impact on adolescents’ school attendance (r=0.416; p<0.00001) (Figure 4).

The odds of a negative impact on adolescents’ physical and social activities and their school attendance were significantly higher in the moderate dysmenorrhea group (OR 3.27, p=0.02; OR 3.51, p=0.01; and OR 3.51, p=0.01, respectively) compared to the mild dysmenorrhea group (Table 2). Additionally, the odds of a negative impact on adolescents’ physical and social activities and their school attendance were significantly higher in the severe dysmenorrhea group (OR 5.6, p=0.0007; OR 19.2, p<0.0001; and OR 6.2, p=0.0001, respectively) compared to the mild dysmenorrhea group (Table 3).

**Table 2:** Odds of negative impact on adolescents` activities and school attendance among adolescents with mild and moderate dysmenorrhea

Variable	Mild dysmenorrhea group (N=60)	Moderate dysmenorrhea group (N=60)	OR [p-value (95%CI)]
**Physical Activities** - Negative effect of dysmenorrhea - Not affected by dysmenorrhea	6 54	16 44	3.27 (0.02* (1.18-9.07))
**Social Activities** - Negative effect of dysmenorrhea - Not affected by dysmenorrhea	7 53	19 41	3.51 (0.01* (1.35-9.14))
**School Attendance** - Negative effect of dysmenorrhea - Not affected by dysmenorrhea	7 53	19 41	3.51 (0.01* (1.35-9.14))

CI: Confidence interval. N: Number. OR: Odd ratio

**Table 3:** Odds of negative impact on adolescents` activities and school attendance among adolescents with mild and severe dysmenorrhea

Variable	Mild dysmenorrhea group (N=60)	Severe dysmenorrhea group (N=60)	OR [p-value (95%CI)]
**Physical Activities** - Negative effect of dysmenorrhea - Not affected by dysmenorrhea	6 54	23 37	5.6 (0.0007* (2.1-15.1))
**Social Activities** - Negative effect of dysmenorrhea - Not affected by dysmenorrhea	7 53	43 17	19.2 (<0.0001* (7.28-50.41))
**School Attendance** - Negative effect of dysmenorrhea - Not affected by dysmenorrhea	7 53	27 33	6.2 (0.0001* (2.4-15.83))

CI: Confidence interval. N: Number. OR: Odd ratio

## DISCUSSION

Primary dysmenorrhea represents a significant challenge for adolescents, often resulting in school absenteeism and impacting their quality of life [7]. Enhancing the quality of life for the younger generation, as well as focusing on disease prevention and early treatment, constitutes a central priority within the healthcare program of the Republic of Kazakhstan for the years 2020-2025 [8]. As a result, we included a total of 180 adolescents aged 12-18 years who were experiencing PD in this cross-sectional comparative study. These participants were categorized into three groups based on their VAS scores: mild dysmenorrhea (VAS ≥1 to ≤3), moderate dysmenorrhea (VAS >3 to ≤7), and severe dysmenorrhea (VAS >7 to ≤10), with the primary objective of assessing how PD impacted the daily lives and school attendance of adolescents.

Our findings revealed a clear and statistically significant positive correlation between the severity of dysmenorrhea and its adverse effects on adolescents' physical activities (p<0.00001) and social activities (p<0.00001). Furthermore, the study demonstrated a significant positive relationship between the severity of dysmenorrhea and its detrimental impact on adolescents' school attendance (p<0.00001). The odds of a negative impact on adolescents’ physical and social activities and school attendance were significantly higher in adolescents experiencing moderate and severe dysmenorrhea than in adolescents with mild dysmenorrhea. This supports previous research by EL-kosery [1], who reported a high rate of class absenteeism due to dysmenorrhea among students, negatively affecting their academic performance. Similarly, a study conducted in a public university in Ethiopia [15] found that dysmenorrhea had a significant negative impact on students’ class attendance, academic performance, and concentration during academic examinations. The study concluded that the educational performance of students with dysmenorrhea was more than eight times negatively affected compared to students without dysmenorrhea [15]. Research by Rafique and Al-Sheikh [6] reported that 8.7% of participants were absent from their academic classes due to dysmenorrhea, and 54.5% of participants indicated a negative impact on their educational performance and daily activities.

Furthermore, Gebeyehu *et al*. [7] found that dysmenorrhea led to social withdrawal, decreased academic performance, restrictions in daily physical activities, and school absenteeism among participants. A significant proportion of participants in their study used home remedies as a primary management approach for dysmenorrhea, with nonsteroidal anti-inflammatory drugs (NSAIDs) being the most commonly used pain relief medication during dysmenorrhea.

Considering the potential risks associated with NSAIDs, such as gastric ulcers and gastrointestinal bleeding [16, 17], alternative therapeutic options for dysmenorrhea relief are valuable. An observational study found that the severity of dysmenorrhea increased in women with low serum vitamin D (Vit. D) levels [18]. A randomized controlled study found that Vit. D was significantly lower in dysmenorrhea, with a significant negative relationship between the severity of dysmenorrhea and Vit. D [19].

A randomized comparative study reported a significant reduction in the severity of dysmenorrhea and the use of NSAIDs after a single oral dose of cholecalciferol (300,000 IU) compared with placebo [20]. Another randomized controlled trial reported that participants who received vitamin D experienced a significant reduction in menstrual pain and consequently reduced their consumption of pain relief medications [4]. Bahrami *et al*. [21] found that high doses of Vit. D (50,000 IU cholecalciferol/week for nine weeks) supplementation reduced the severity of dysmenorrhea. Additionally, a systematic review reported an inverse relationship between serumVit. D and the severity of dysmenorrhea [22].

### Relationship between dysmenorrhea and BMI

The correlation between BMI and PD remains a subject of debate [23]. Ju *et al*. [24] observed an increased risk of dysmenorrhea in both underweight and obese individuals, while Jiang *et al*. [25] reported an increased odds of dysmenorrhea in women with either lower or higher BMI. Furthermore, Gurdip *et al*. [26] identified a significant relationship between dysmenorrhea and underweight or overweight women. The controversial relationship between BMI and PD explains why underweight and overweight adolescents were excluded from this study. To address this relationship, further studies are required.

This study was the first comparative study conducted in West Kazakhstan (Aktobe) to detect the impact of primary dysmenorrhea on adolescents’ activities and school attendance following the health care program of the Republic of Kazakhstan. In this study, there was a significant positive relationship between the severity of dysmenorrhea and its negative impact on adolescents’ physical and social activities. Additionally, this study found a significant positive relationship between the severity of dysmenorrhea and its negative impact on adolescents’ school attendance. The odds of a negative impact on adolescents’ physical and social activities and school attendance were significantly higher in adolescents suffering from moderate and severe dysmenorrhea than in adolescents with mild dysmenorrhea. Adolescents' refusal to participate was one of the limitations of this study. Moreover, the study was conducted exclusively in West Kazakhstan (Aktobe), which limits the generalizability of the findings to a broader population. Future studies are essential to establish a more comprehensive understanding of the impact of PD on adolescents' activities and school attendance.

This study recommends implementing a national health education program to increase awareness among school and university authorities regarding the negative impact of dysmenorrhea on adolescents’ academic performance. Furthermore, it should aim to increase the awareness of adolescents and their families regarding the benefits of maintaining an ideal BMI and normal serum vitamin D levels.

## CONCLUSION

The odds of a negative impact on adolescents’ physical and social activities and school attendance were significantly higher in adolescents experiencing moderate and severe dysmenorrhea than in adolescents with mild dysmenorrhea. This study suggests a national health education program aimed to increase awareness among school and university authorities regarding the negative impact of dysmenorrhea on adolescents’ academic performance and among adolescents and their families regarding the benefits of maintaining ideal BMI and normal serum Vit. D.
